# The Cyanobacterium *Cylindrospermopsis raciborskii* (CYRF-01) Responds to Environmental Stresses with Increased Vesiculation Detected at Single-Cell Resolution

**DOI:** 10.3389/fmicb.2018.00272

**Published:** 2018-02-21

**Authors:** Victor Zarantonello, Thiago P. Silva, Natália P. Noyma, Juliana P. Gamalier, Mariana M. Mello, Marcelo M. Marinho, Rossana C. N. Melo

**Affiliations:** ^1^Laboratory of Cellular Biology, Department of Biology, Federal University of Juiz de Fora, Juiz de Fora, Brazil; ^2^Laboratory of Ecology and Physiology of Phytoplankton, Department of Plant Biology, Rio de Janeiro State University, Rio de Janeiro, Brazil; ^3^Laboratory of Aquatic Ecology, Department of Biology, Federal University of Juiz de Fora, Juiz de Fora, Brazil

**Keywords:** outer membrane vesicles, extracellular vesicles, cyanobacteria, *Cylindrospermopsis raciborskii*, ultraviolet radiation, interspecific interaction, transmission electron microscopy, phosphatidylserine

## Abstract

Secretion of membrane-limited vesicles, collectively termed extracellular vesicles (EVs), is an important biological process of both eukaryotic and prokaryotic cells. This process has been observed in bacteria, but remains to be better characterized at high resolution in cyanobacteria. In the present work, we address the release of EVs by *Cylindrospermopsis raciborskii* (CYRF-01), a filamentous bloom-forming cyanobacterium, exposed to environmental stressors. First, non-axenic cultures of *C. raciborskii* (CYRF-01) were exposed to ultraviolet radiation (UVA + UVB) over a 6 h period, which is known to induce structural damage to this species. Second, *C. raciborskii* was co-cultured in interaction with another cyanobacterium species, *Microcystis aeruginosa* (MIRF-01), over a 24 h period. After the incubation times, cell density and viability were analyzed, and samples were processed for transmission electron microscopy (TEM). Our ultrastructural analyses revealed that *C. raciborskii* constitutively releases EVs from the outer membrane during its normal growth and amplifies such ability in response to environmental stressors. Both situations induced significant formation of outer membrane vesicles (OMVs) by *C. raciborskii* compared to control cells. Quantitative TEM revealed an increase of 48% (UV) and 60% (interaction) in the OMV numbers compared to control groups. Considering all groups, the OMVs ranged in size from 20 to 300 nm in diameter, with most OMVs showing diameters between 20 and 140 nm. Additionally, we detected that OMV formation is accompanied by phosphatidylserine exposure, a molecular event also observed in EV-secreting eukaryotic cells. Altogether, we identified for the first time that *C. raciborskii* has the competence to secrete OMVs and that under different stress situations the genesis of these vesicles is increased. The amplified ability of cyanobacteria to release OMVs may be associated with adaptive responses to changes in environmental conditions and interspecies cell communication.

## Introduction

The bloom-forming cyanobacterium *Cylindrospermopsis raciborskii* has attracted considerable attention due its widespread distribution and its potential ability to produce diverse toxins, such as hepatotoxins and neurotoxins that can cause detrimental impacts on the environmental health (Molica et al., 2002; Carneiro et al., 2013). Some cyanotoxins are directly associated with fish, domestic livestock, and even human mortalities (Codd et al., 2005; Dittmann and Wiegand, 2006; Svircev et al., 2016). The mechanisms that govern the geographic expansion and bloom formation of *C. raciborskii* involve its high plasticity and physiological tolerance to a wide range of environmental conditions, such as light, nutrients and temperature, as well as its antagonistic interactions with other phytoplankton species (Padisák, 1997; Beamud et al., 2016; Burford et al., 2016). Furthermore, production of allelochemicals by *C. raciborskii* has been proposed as an additional advantage to the dominance of this species *via* inhibition of other cyanobacteria competitors, including *Microcystis aeruginosa* (Figueredo et al., 2007; Mello et al., 2012).

Secretion of membrane-limited vesicles, collectively termed extracellular vesicles (EVs), is an important cellular event of both eukaryotic and prokaryotic cells. The capacity of bacteria to release EVs has been exponentially demonstrated (reviewed in Kulp and Kuehn, 2010; Jan, 2017). These nano-scale vesicles are extruded from the outer membrane of gram-negative bacteria and have been associated with fundamental biological processes such as pathogenesis (Kolling and Matthews, 1999; Rivera et al., 2010); cellular defense (Manning and Kuehn, 2011; Baumgarten et al., 2012), cell communication (Mashburn-Warren et al., 2008) and DNA transfer (Rumbo et al., 2011). In marine microbial communities, cyanobacteria and heterotrophic bacteria-derived vesicles were reported to be abundant in coastal and open-ocean seawater samples and implicated in marine carbon flux (Biller et al., 2014). More recently, it was indicated that the cyanobacterium *Synechocystis* PCC6803 produces EVs in a manner similar to gram-negative bacteria, that is, from the outer membrane (Pardo et al., 2015). However, little is still known on the ability of cyanobacteria to secrete outer membrane vesicles (OMVs), their biogenesis and potential functions in aquatic biology.

It has been reported that bacteria produce EVs in freshwater ecosystems (Silva et al., 2014) and that ultraviolet (UV) radiation, an environmental stressor, elicits increased vesiculation in heterotrophic bacteria from freshwater (Gamalier et al., 2017). The amplified release of EVs might be a regulated response offering an effective adaptive mechanism under natural and diverse stress conditions (reviewed in Jan, 2017). EVs could pack a variety of cargos related to population survival and persistence, removal of undesirable envelope proteins (McBroom and Kuehn, 2007) or self-defense molecules (Rivera et al., 2010; Manning and Kuehn, 2011).

In the present work, we tested the hypothesis that the cyanobacterium C. *raciborskii* is also able to produce EVs and increases this ability as an adaptive mechanism to underlie the responses to different kinds of environmental stressors such as UV radiation and interaction with *M. aeruginosa*. UV radiation affects *C. raciborskii* cells leading to molecular and structural changes (Noyma et al., 2015) while the interspecific interaction between *C. raciborskii* and *M. aeruginosa* promotes mutual antagonistic effects through allelopathy (Figueredo and Giani, 2009; Mello et al., 2012; Rzymski et al., 2014).

The use of transmission electron microscopy (TEM) enables unambiguous visualization of EVs in both eukaryotic and prokaryotic cells, and their genesis during different situations (Akuthota et al., 2016; Gamalier et al., 2017). By performing a comprehensive TEM study, we demonstrated, for the first time, that *C. raciborskii* produces EVs, which were clearly characterized as typical nanovesicles shedding out from the outer membrane. Quantitative TEM revealed that both environmental UV and interspecific interaction were able to positively influence the biogenesis of OMVs by this cyanobacterium.

## Materials and Methods

### Cyanobacterial Strains and Stock Cultures

Strains of *C. raciborskii* (CYRF-01) and *M. aeruginosa* (MIRF-01) were obtained from the cyanobacteria culture collection of the Laboratory of Cyanobacterial Ecophysiology and Toxicology, Federal University of Rio de Janeiro (LETC) (Brazil). Both strains were isolated from Funil Reservoir (Brazil) in 2005, where these species act as natural competitors and show seasonal dominance (Soares et al., 2009). In cultures, *C. raciborskii* (CYRF-01) grows as filamentous colonies while *M. aeruginosa* (MIRF-01) grows predominantly as single cells (Bolch and Blackburn, 1996). Usually, CYRF-01 produces saxitoxins, whereas MIRF-01 produces microcystins (Bláha et al., 2009; Ferrão-Filho et al., 2009; Mello et al., 2012). Both strains were maintained in sterile ASM-1 growth medium (Gorham et al., 1964) in 300 mL Erlenmeyer flasks placed in a climate-controlled room at 25°C, 35 μmol photons m^-2^s^-1^, with a photoperiod of 12:12 h (Mello et al., 2012).

### UV Exposure

In order to evaluate the production of EVs by *C. raciborskii* in response to UV radiation, we exposed cultures to artificial UV radiation (UVA + UVB, 280–400 nm) as described in a previous work (Noyma et al., 2015). Briefly, samples of *C. raciborskii* from cultures in exponential growth phase were re-suspended in 40 mL of fresh ASM-1 medium (Gorham et al., 1964) at an initial concentration of 10^6^ cells/mL and were exposed to artificial UV radiation supplied by UVA (TL 40/05; Philips; emission peak at 365 nm) and UVB (TL 20/01; Philips; emission peak at 312 nm) lamps (Noyma et al., 2015). The UV doses used in the experiment were 254.88 KJ m^2^ (UVA) and 11.66 KJ m^2^ (UVB) while the intensities were 11.8 W m^-2^ (UVA) and 0.54 W m^-2^ (UVB). The control treatment was performed in quartz flasks (40 mL) under photosynthetically active radiation (PAR; 400–700 nm) at 90 μmol photons m^-2^ s^-1^. Treatments were performed during 6 h at room temperature (RT; 20 ± 1°C) and all samples were carefully homogenized prior to subsequent analyses. All experiments were performed in triplicate.

### Cyanobacteria Species Interaction

To investigate the potential vesiculation process in *C. raciborskii* in response to an interspecific interaction, we used as a model the co-culture of the strains CYRF-01 and MIRF-01. Monocultures of CYRF-01 and mixed cultures with 50% CYRF-01/50% MIRF-01 at a concentration of 10^6^ cells/mL were established from stock cultures as before (Mello et al., 2012). Mono and mixed cultures were maintained in 125 mL Erlenmeyer flasks containing 40 mL of ASM-1 growth medium at the same controlled conditions (25°C and 55 μmol photon m^-2^s^-1^, photoperiod 12:12 h). There was no resource limitation during the experiment period. After 24 h, aliquots from *C. raciborskii* monocultures (controls) and mixed cultures were taken for subsequent analyses. All experiments were performed in triplicate.

### Cell Density

Samples were taken from each experimental group for cell density determination as before (Sipaúba-Tavares and Rocha, 2003). Samples were fixed with lugol solution for preservation and the cells were counted on a light microscope (B × 41, Olympus) at 400 × magnification by using an improved Neubauer hemocytometer (Sipaúba-Tavares and Rocha, 2003). Analyses were performed at the beginning (0 h) and at the end of UV (6 h) and interaction (24 h) experiments.

### Cyanobacterial Cell Viability

The formation of EVs may be associated with cell death/viability (Gamalier et al., 2017). Cell membrane integrity was investigated by using a fluorescent probe (Backlight) as an indicator for cell viability (Boulos et al., 1999). Samples were collected from each group and the proportion of live/viable and dead/non-viable cells was determined using LIVE/DEAD BacLight viability kit (Molecular Probes, Inc, Thermo Fisher Scientific, Eugene, OR, United States). This kit contains a mixture of fluorescent dyes, SYTO^®^ 9 and propidium iodide, which differ both in their spectral characteristics and their ability to penetrate healthy bacterial cell membranes. Cells with intact membranes (live cells) stain green and those with damaged membranes (dead cells) stain red (Barbesti et al., 2000; Joux and Lebaron, 2000; Hoefel et al., 2003; Berney et al., 2007). A mixture of equal volumes of the stains (total volume of 0.9 μL) was added to 300 μL of each sample and incubated for 20 min in the dark. Slides (*n* = 3) for each time point were prepared in a cytocentrifuge (Shandon Cytospin 4, Thermo Electron) as before (Silva et al., 2014; Noyma et al., 2015) at 28 × *g* during 5 min at medium acceleration and evaluated under a fluorescence microscope (BX-60, Olympus, Melville, NY, United States) at 450–480 nm excitation wavelengths, which enable simultaneous visualization of live and dead cells. For each group, 30 filaments were counted and the percentage of live/dead cells determined. Images were taken using Evolution VF (Media Cybernetics, Silver Spring, MD, United States) digital camera and Image Pro-Plus 5.0 software (Media Cybernetics, Silver Spring, MD, United States).

### Transmission Electron Microscopy (TEM)

*Cylindrospermopsis raciborskii* samples collected from treated groups and respective controls were immediately fixed in a mixture of freshly prepared aldehydes (1% paraformaldehyde and 1% glutaraldehyde) in 0.1 M phosphate buffer, pH 7.3, for 1 h at RT before any subsequent centrifugation procedure. Next, samples were washed twice in 0.1 M phosphate buffer, pH 7.3 (3,000 × *g* for 10 min), and stored at 4°C for subsequent use. After fixation, agar embedding was performed as before (Silva et al., 2014; Noyma et al., 2015), so that uniformly distributed specimens could be processed as easily handled blocks of cells. Agar pellets containing water specimens were post-fixed in a mixture of 1% phosphate-buffered osmium tetroxide and 1.5% potassium ferrocyanide (final concentration) for 1 h. After washing with 0.1 M phosphate buffer, pH 7.3, pellets were stained *en bloc* in 2% uranyl acetate in 0.1 M phosphate buffer, pH 7.3 at RT and washed in the same buffer prior to dehydration in graded ethanols (70, 95, and 100%), and infiltration and embedding in a propylene oxide-Epon sequence (PolyBed 812, Polysciences, Warrington, PA, United States) (Silva et al., 2014). After polymerization at 60°C for 16 h, thin sections were cut using a diamond knife on an LKB ultramicrotome (LKB Instruments, Gaithersburg, MD, United States). Sections were mounted on uncoated 200-mesh copper grids (Ted Pella) before staining with lead citrate and cyanobacteria were examined using a transmission electron microscope (Tecnai Spirit G12; FEI Company, Eindhoven, Netherlands) at 80 kV.

### Quantitative TEM Analyses

To perform a quantitative analysis of EVs, electron micrographs were randomly taken at magnifications of 30,000–75,000×. A total of 122 electron micrographs (UV group: 40 cell sections; interspecific interaction group: 42 cell sections; control groups: 40 cell sections) and a total of 396 OMVs were analyzed. Then, the number of EVs/cell section and the proportion of *C. raciborskii* releasing vesicles were established, as well as the number of OMVs in process of outward budding or closely associated with the cell surface. Additionally, the diameters of EVs were measured and grouped in different ranges (20–80, 81–140, 141–200, 201–260, and 261–320 nm). These analyses were done in clear cell sections exhibiting intact cell envelopes and each cell on a filament was considered separately. All quantitative studies were performed using the *Image J* software (National Institutes of Health, Bethesda, MD, United States).

### Annexin V Analysis

In eukaryotic cells, it is recognized that phosphatidylserine is relocated to the plasma membrane leaflet at sites on the cell surface where EV shedding occurs (reviewed in Hugel et al., 2005; Muralidharan-Chari et al., 2010). To detect exposed phosphatidylserine, cells were stained with FITC-conjugated Annexin V, a marker for this molecule (Invitrogen, Carlsbad, CA, United States). Samples (1 × 10^7^ cells/mL) were collected, washed in phosphate-buffered saline (PBS) (0.1 M sodium phosphate, 0.15 M sodium chloride, pH 7.4) and stained with annexin -V - FITC (20 μM) (Dwyer et al., 2012). After incubation for 20 min, samples were analyzed by both differential interference contrast (DIC) and fluorescence microscopy (BX-60, Olympus, Melville, NY, United States) at 450–480 nm excitation wavelengths (FITC). For each group, 30 filaments were counted and the percentage of annexin-positive cells determined. Experiments were performed in triplicate.

### Statistical Analyses

Data from cyanobacteria cell density and viability analyses were compared using ANOVA, followed by the Turkey’s comparison test. OMVs number by TEM analyzes and proportion of annexin-V positive cells by fluorescence microscopy were compared using the Student’s *t*-test (*P* < 0.05). Statistical analyses and graphs were performed using the software Prism 6.0.1 (GraphPad software, San Diego, CA, United States).

## Results

### *C. raciborskii* Releases EVs

**FIGURE 1:**
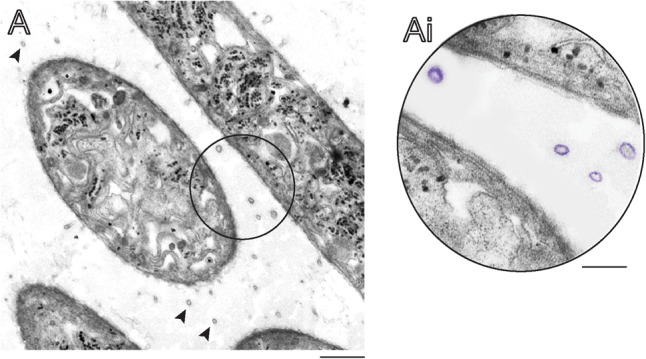
Longitudinal and cross sections of *Cylindrospermopsis raciborskii* growing in control conditions seen by transmission electron microscopy (TEM). **(A)** Several extracellular membrane vesicles (indicated by arrowheads in **A** and highlighted in purple in **Ai**) are observed around cultured cyanobacterial cells.

**FIGURE 2:**
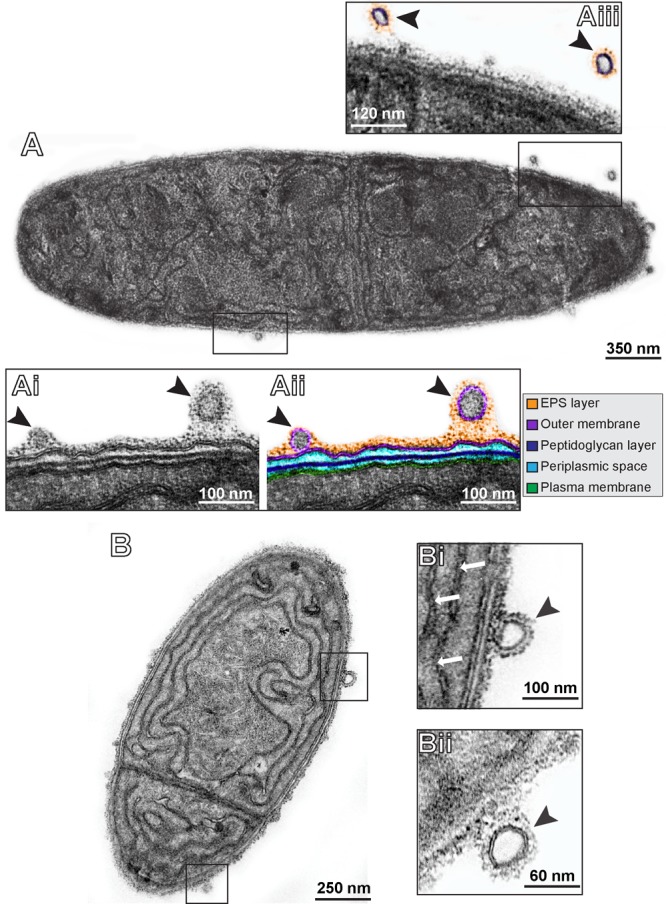
Representative electron micrographs of *C. raciborskii* cells under control culture conditions **(A,B)**. In **(Ai,Aii)**, the cell envelope is seen in high magnification. This structure is composed of two bilayered membranes: the inner or plasma membrane (highlighted in green) and the outer membrane (highlighted in purple) that encloses the periplasmic space (light blue) with a thin peptidoglycan layer (dark blue). Note the presence of OMVs (arrowheads) with typical trilaminar structure cleary budding off from the outer membrane. Secreted vesicles frequently exhibited an external amorphous material (extracellular polymeric substances – EPS) as observed on the surface of the cell envelope (highlighted in orange in **Aii,Aiii**). Thylacoid membranes are indicated by white arrows in **(Bi)**.

Over the past years, our research group has been studying the ultrastructure of bacteria and cyanobacteria by TEM both *in situ* and in cultures (Silva et al., 2014, 2016; Noyma et al., 2015; Gamalier et al., 2017). Our EM methodology includes prompt aldehyde fixation while the cells are still in suspension and before any subsequent centrifugation procedure, which is important to optimal cell preservation and to capture specific biological events in response to varied stimuli. By examining resulting electron micrographs from *C. raciborskii* growing in control culture conditions, small vesicles were found closely associated with this cyanobacterium (**Figure 1**). Similar to other cyanobacteria, this species is characterized by an envelope composed of three layers: an inner (plasma membrane), an intermediate (periplasmic space and peptidoglycan layer) and an outer membrane (**Figures 2Ai,Aii**). Our ultrastructural analyses clearly revealed vesicles budding off from the outer membrane of *C. raciborskii* cells (**Figure 2**). These OMVs were identified as round vesicles with a trilaminar structure typical of bilayered phospholipidic membranes (**Figures 2Bi,Bii**). Moreover, secreted vesicles frequently exhibited an external amorphous coating as observed on the surface of the cell envelope (extracellular polymeric substances - EPS) (**Figure 2Ai**). We found that 89.8 ± 5.9 % (mean ± SEM, *n* = 396 vesicles) of OMVs exhibited this EPS layer.

### Production of OMVs by *C. raciborskii* Increases with UV Radiation-Induced Stress

**FIGURE 3:**
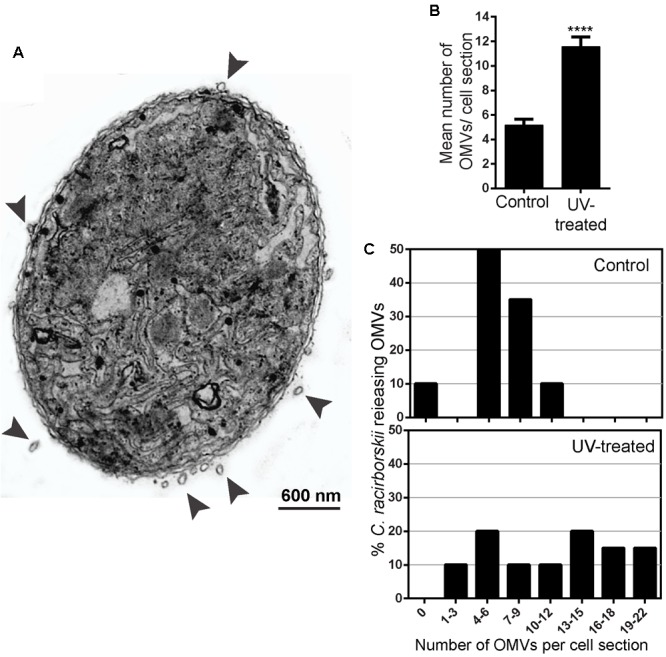
Release of OMVs by *C. raciborskii* increases in response to UV radiation. In **(A)**, a representative electron micrograph of an UV-exposed *C. raciborskii* cell shows several OMVs (arrowheads) in different degrees of extrusion from the cyanobacterial surface. OMVs per cell section and the proportion of cells releasing OMVs are shown in **(B)** and **(C)**, respectively. Note that most cells (70%) produced 7–22 OMVs/cell section in the UV-treated group while 50% of the cells produced 4–6 OMVs/cell section in the control group. Counts were derived from three experiments with a total of 396 OMVs counted in 122 electron micrographs. ^∗∗∗∗^*P* < 0.0001.

We next investigated the production of OMVs by *C. raciborskii* in response to UV radiation, as observed before for bacteria from aquatic ecosystems (Gamalier et al., 2017). After we treated cultures of *C. raciborskii* with UV as done previously (Noyma et al., 2015), TEM micrographs showing intact plasma membrane were randomly taken and carefully examined. Exposure to this radiation triggered the release of OMVs by *C. raciborskii* (**Figure 3A**). To quantify the number of OMVs from the experimental and control groups, *C. raciborskii* cell sections were evaluated (*n* = 40 cells), and a total of 164 OMVs were counted. UV radiation led to a significant increase of OMV production (11.56 ± 3.34 OMVs/cell section,) compared to control cells (5.16 ± 1.67 OMVs/cell section, *P* < 0.0001) (**Figure 3B**). Moreover, by scoring the number of vesicles, we found that in untreated cells, 50% of OMV-producing cells released 4–6 OMVs/cell section whereas 70% of UV-treated cells produced 7–22 OMVs/cell section (**Figure 3C**).

### *C. raciborskii* Vesiculation Increases in Response to Interaction with *M. aeruginosa*

**FIGURE 4:**
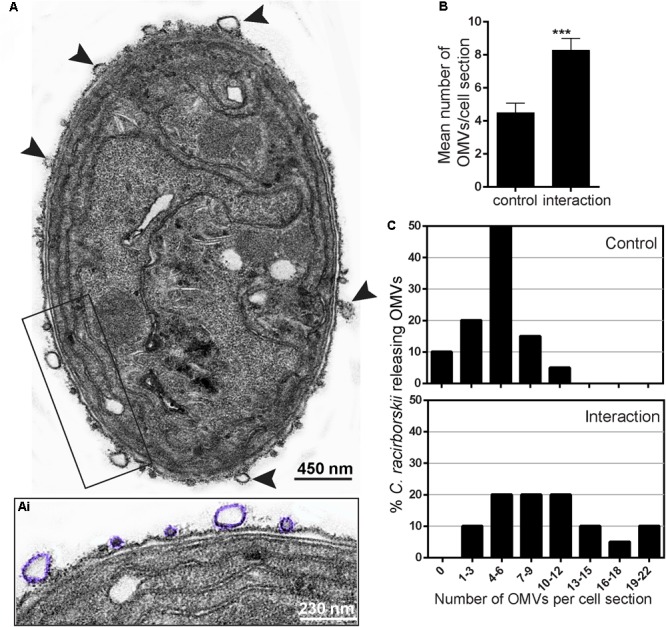
Co-culture with a *Microcystis aeruginosa* strain induces vesiculation by *C. raciborskii*. In **(A)**, a representative electron micrograph of a *C. raciborskii* cell, collected from a mixed culture with *M. aeruginosa*, shows several OMVs (arrowheads) at the cell surface. Note the different sizes of nascent OMVs (highighted in purple in **Ai**). The total number of OMVs per cell section and the proportion of cells releasing OMVs are shown in **(B)** and **(C)**, respectively. While most *C. raciborskii* cells (65%) produced 7–22 OMVs/cell section in the mixed culture, most cells (50%) in the control group released 4–6 OMVs/cell section. Counts were derived from three experiments with a total of 396 OMVs counted in 122 electron micrographs. ^∗∗∗^*P* < 0.0001.

We also detected an increased vesiculation by *C. raciborskii* when cultivated with a *M. aeruginosa* strain (**Figures 4A,Ai**). Quantitative EM analysis showed significant increase in the total numbers of OMVs secreted by *C. raciborskii* in co-cultures (8.27 ± 3.07 OMVs/ cell section) compared to controls in monocultures (4.49 ± 1.98 OMVs/cell section, *P* < 0.001) (**Figure 4B**). By scoring the numbers of OMVs, we found that while 50% of *C. raciborskii* in monocultures released 4–6 OMVs/cell section, 65% of this species in co-cultures produced 7–22 vesicles/cell section (**Figure 4C**).

### *C. raciborskii* Vesiculation Is a Dynamic Process

**FIGURE 5:**
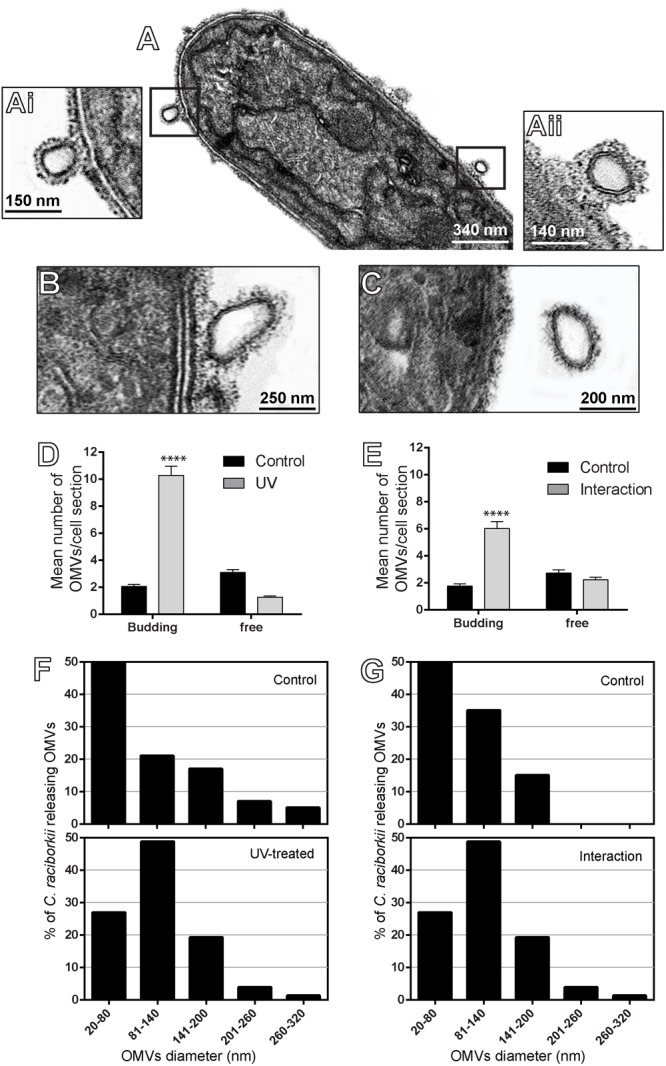
Characterization of nascent OMVs and budding rate from *C. raciborskii* cells. In **(A–C)**, OMVs with different sizes and in progressive outward budding from the cell surface are observed. **(Ai,Aii)** Are boxed areas of **(A)** seen at higher magnification. **(D,E)** The numbers of budding OMVs significantly increased in response to both UV radiation **(D)** and interaction with *M. aeruginosa* **(E)**, compared with respective controls. **(F,G)** Both treatments elicited secretion of OMVs with higher size in comparison with controls. Counts were derived from three experiments with a total of 396 OMVs counted in 122 electron micrographs. ^∗∗∗∗^*P* < 0.0001.

In eukaryotic cells, the genesis of EVs is a rapid event with nascent vesicles being observed by TEM in different stages of outward budding from the plasma membrane and/or completely released at the cell surface (Akuthota et al., 2016). We next studied in more detail the process of vesicle release in *C. raciborskii* by quantitative evaluation of nascent OMVs that were detaching from the outer membrane (budding OMVs) or free at the cell surface (**Figures 5A–C**). The numbers of budding OMVs in *C. raciborskii* cells were significantly higher in both treated groups (**Figures 5D,E**) compared to the controls.

**FIGURE 6:**
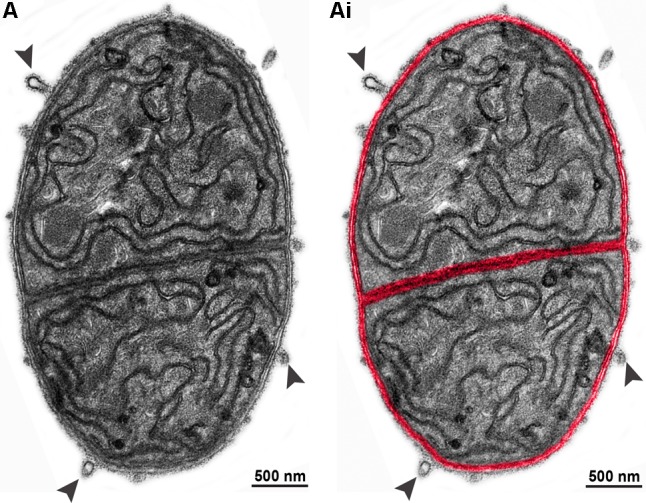
Vesiculation as a dynamic event in *C. raciborskii* cells in the same filament. **(A)** Electron micrograph from a representative *C. raciborskii* filament after UV-exposure shows two cells in process of vesiculation. Note the high number of OMVs (indicated by arrowheads) being formed at the cell surface. In **(Ai)**, the cell envelope was colored in red to highlight the contours of individual cells.

In addition, we also established the average size of OMVs released from *C. raciborskii* cells to be 86.23 ± 4.86 nm (mean ± SEM) in diameter in control conditions and 99.64 ± 4.53 and 101.50 ± 4.62 (mean ± SEM) nm after UV exposure and interaction with *M. aeruginosa*, respectively. Considering all conditions (control and treated groups), OMV diameters varied from 20 to 320 nm, with most OMVs showing diameters between 20 and 140 nm (**Figures 5F,G**). OMVs released in response to stress stimulus (UV exposure and species interaction) were larger compared to controls (**Figures 5F,G**). Interestingly, our TEM analyses enabled to detect vesiculation by individual cells closely interrelated in the same filament (**Figure 6**).

### Phosphatidylserine Relocates in *C. raciborskii* Outer Membrane

**FIGURE 7:**
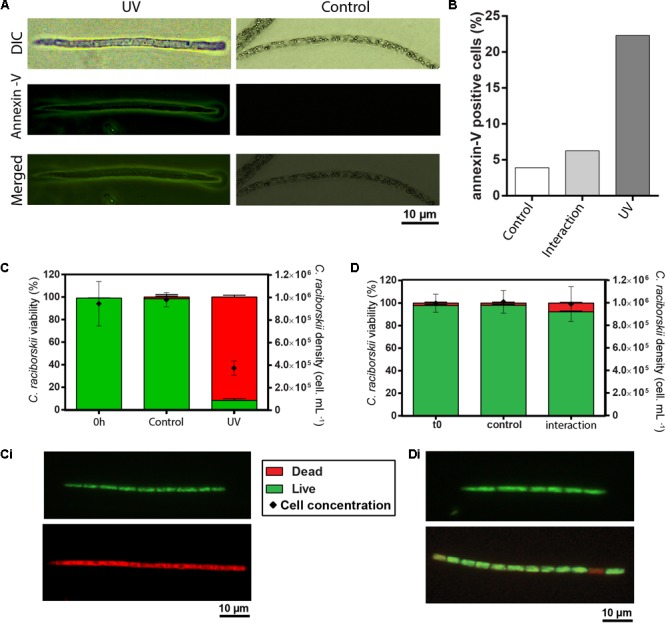
Phosphatidylserine relocation, cell density and cell viability analyses of *C. raciborskii.* **(A)** Representative images of *C. raciborskii* cells seen by differential interference contrast (DIC) and fluorescence microscopy (identical fields) after exposure or not to UV-radiation. Green fluorescence indicates annexin-V-positive cells. **(B)** The proportion of annexin-V-positive cells increased after exposure to both UV radiation and interaction with *M. aeruginosa*. **(C,D)** *C. raciborskii* density and viability significantly decreased in response to UV radiation **(C)** but not in mixed cultures with *M. aeruginosa* **(D)**. **(Ci,Di)** Show representative fluorescent images from cells stained with Baclight. Live cells are seen in green and dead cells in red.

As noted, phosphatidylserine relocation may be associated with EV shedding (reviewed in Hugel et al., 2005; Muralidharan-Chari et al., 2010). Thus, we studied whether the same phenomenon would happen in the outer membrane of cyanobacteria. We stained cells with annexin-V-FITC and samples were analyzed by fluorescence microscopy (**Figure 7A**). A higher proportion of *C. raciborskii* showed annexin-V-positive staining after both UV treatment and interaction with *M. aeruginosa* compared to control cells (**Figure 7B**).

### Cell Viability and Density of *C. raciborskii* upon UV Radiation Exposure and Interaction with *M. aeruginosa*

Because annexin-V is also used as a marker for cell death, we also evaluated both the cell concentration and cell viability by using classical assessments for cell density (DAPI staining) and membrane viability (baclight) (Joux and Lebaron, 2000; Sipaúba-Tavares and Rocha, 2003). As expected, the cell density was significantly reduced while the proportion of non-viable cyanobacteria significantly increased after UV exposure (**Figures 7C,Ci**), as previously demonstrated (Noyma et al., 2015). On the other hand, neither reduction of the cell density nor loss of the cell viability was observed as a result of the interaction with *M. aeruginosa* (**Figures 7D,Di**).

## Discussion

The data presented in this work demonstrate, for the first time, that vesiculation is a common phenomenon for *C. raciborskii*. By studying the strain CYRF-01, we showed that this cyanobacterium constitutively releases OMVs during its normal growth and amplifies such ability in response to environmental stressors, such as UV radiation and interaction with a potential competitor. We thus recognized that *C. raciborskii* has the competence to secrete OMVs and deals with different stress situations with increased genesis of these vesicles. Our data are supported by previous works on other cyanobacterial species and different strains which found production of EVs in response to stressors such as hyperosmotic (hypersaline) conditions (Xu et al., 2013), antibiotic supplementation (Oliveira et al., 2016) or reduced temperature (Oliveira et al., 2016). Moreover, the present work expands our understanding of cyanobacteria as organisms able to actively release EVs (Zheng et al., 2009; Biller et al., 2014; Oliveira et al., 2015; Pardo et al., 2015; Brito et al., 2017).

To identify and characterize EVs, we used TEM, a technique that distinguishes EVs from non-membranous particles of similar size (Lotvall et al., 2014). This is because EVs are nano-structures delimited by a phospholipid membrane, which can be unambiguously imaged by TEM as a “trilaminar” structure in which the hydrophilic phosphate “heads” are electron-dense and the hydrophobic fatty acids “tails” are electron-lucent (Bozzola and Russell, 1999; Lodish et al., 2016). For this reason, the use of TEM provides the most direct evidence for EV production. Indeed, TEM is considered a gold standard technique to characterize individual EVs (Akuthota et al., 2016; Lawson et al., 2016; Gamalier et al., 2017) as endorsed by the International Society for Extracellular Vesicles (ISEV) (Lotvall et al., 2014). Moreover, the ISEV recommends that, for better characterization of the vesiculation event, TEM images should show a “wild field” encompassing multiple vesicles in addition to close-up images of single vesicles at cell surface (Lotvall et al., 2014). Our TEM analyses included a quantitative approach, which enabled clear size characterization and quantification of many nascent EVs at the entire cell surface (**Figures 2–4, 6**), providing conclusive evidence for the production of EVs by *C. raciborskii*. We also provided, for the first time, direct evidence that EVs released by this cyanobacterium are formed by fission from the outer membrane, similar to the process described for liberation of OMVs by gram-negative bacterial species (reviewed in Kulp and Kuehn, 2010; Jan, 2017). That cyanobacteria vesiculate similar to gram-negative bacteria was suggested by previous studies (Zheng et al., 2009; Oliveira et al., 2015; Brito et al., 2017). In a study using *Synechocystis* PCC6803, for example, proteins and lipids derived from the outer membrane were detected in cell-free supernatants derived from *Synechocystis* (Pardo et al., 2015). Additionally, our high-resolution approach enabled imaging of the EPS layer in most nascent OMVs. This means that OMVs present a layer of polysaccharidic nature, which might provide structural integrity and protection against UV radiation, important for persistence of OMVs in the environment (Pereira et al., 2009). Other potential roles for the EPS layer in OMVs include serving as an alternative storage for carbon compounds (Pereira et al., 2009; Pannard et al., 2016). In fact, sugars are molecules previously hypothesized to be transported by OMVs in cyanobacteria (Xu et al., 2013).

Our study also demonstrated that the number of OMVs in process of outward budding from the outer membrane, that is, still physically attached to this membrane, increased in cells under stress compared to cells in normal growth (**Figure 5**). The higher rate of OMV production after exposure to the stress conditions possibly enabled us to capture more frequently the different degrees of vesicle budding off from the outer membrane. Moreover, our findings revealed that nascent OMVs show varied sizes (range of 20–320 nm, **Figure 5**), indicating that there are morphologically different populations of OMVs secreted by *C. raciborskii.* We also captured the vesiculation event by individual cells in the same filament (**Figure 6**) indicating that different cells are able to respond collectively to the stressor agents.

To explore the link between vesicle release and environmental stress, we exposed *C. raciborskii* to different conditions, such as UV radiation, which is known to promote clear alterations in the density, viability and photosynthetic structures of this cyanobacterium (Noyma et al., 2015) as well as damage in other species of cyanobacteria (reviewed in Singh et al., 2010). Our findings showed an increased production of OMVs in response to UV radiation. Remarkably, our analyses at high resolution also showed that vesiculation by UV-treated cyanobacteria were not a result of cell lysis. Although most cells were non-viable, as detected by a marker of membrane permeability (**Figure 7**), nascent OMVs were released from structurally intact cyanobacteria (**Figures 3, 5**). The same phenomenon was observed for gram-negative bacteria exposed to UV radiation (Gamalier et al., 2017) indicating that damaged cells release OMVs before cell lysis likely as a protective mechanism against UV radiation to increase cell survival since cell compounds such as DNA could be preserved within these nanovesicles.

When in co-cultures with *M. aeruginosa, C. raciborskii* can inhibit the growth of *M. aeruginosa* strains (Figueredo et al., 2007; Mello et al., 2012; Rzymski et al., 2014). Allelochemicals secreted by *C. raciborskii* are likely mediating this action (Figueredo et al., 2007; Rzymski et al., 2014). Because EVs constitute a way by which both eukaryotic and bacterial cells secrete products to the extracellular medium, we hypothesized that *C. raciborskii* would be able to increase vesiculation upon interaction with *M. aeruginosa*. Our results clearly demonstrated such capacity. Although the chemical identity of the cargo within the vesicles remains to be established, it is clear that *C. raciborskii* responds to the interaction with production of secretory vesicles, which might be involved in the mechanism for *M. aeruginosa* growth inhibition.

Phosphatidylserine is an anionic glycerophospholipid present in the membranes of both eukaryotic (reviewed in Kay and Grinstein, 2011) and some prokaryotic cells (reviewed in Sohlenkamp and Geiger, 2016). In eukaryotes, phosphatidylserine is located on the internal leaflet of the plasma membrane and its externalization, that is, its exposure on the outer leaflet of this membrane, has been reported to be a distinct molecular event during formation of EVs (reviewed in Hugel et al., 2005; Muralidharan-Chari et al., 2010), being thus used as an additional evidence for cell vesiculation (Gonzalez-Cano et al., 2010; Akuthota et al., 2016). Here, we demonstrated that *C. raciborskii* filaments under stress conditions have higher proportion of phosphatidylserine-positive cells compared to cells growing in control conditions (**Figure 7**). Phosphatidylserine externalization is also an event commonly associated with apoptosis in both prokaryotic (reviewed in Zheng et al., 2013; Kasuba et al., 2015) and eukaryotic (reviewed in Suzanne and Steller, 2013) cells. However, overall, our findings indicate that phosphatidylserine externalization can be mostly considered another evidence for *C. raciborskii* vesiculation instead of an indicative of cell death. First, by using a marker for cell viability, both non-viable/dead and viable/live cells, evaluated after UV exposure or interspecific interaction showed phosphatidylserine externalization (**Figure 7**). Second, TEM, which is considered a gold standard to detect apoptosis, revealed absence of typical or similar morphological features of apoptosis in *C. raciborskii* cells after both treatments, such as the occurrence of empty cells with intact cell envelope and/or cells with cytoplasmic condensation/retraction (reviewed in Silva et al., 2017). Therefore, the phosphatidylserine analyses corroborate our TEM results showing amplified *C. raciborskii* vesiculation in response to stress conditions.

Our phosphatidylserine findings also highlight an important biological aspect of *C. raciborskii*: this cyanobacterium appears to change the composition/organization of its membrane lipids in response to alterations in the environment as documented for other bacteria (reviewed in Sohlenkamp and Geiger, 2016). In fact, different bacterial species are able to change membrane lipid composition/organization/fluidity to survive under unfavorable conditions or even to adapt to a new situation such as nutrient deprivation or increase/decrease of temperature (Sohlenkamp and Geiger, 2016). In addition to underlie the mechanism of OMV production, membrane lipid modification in *C. raciborskii* could allow a rapid response to changes in environmental conditions, as observed for other bacterial species (Sohlenkamp and Geiger, 2016).

Taken together, our findings identify, for the first time, that *C. raciborskii* (CYRF-01) secretes OMVs during normal growth and that the release of these vesicles to the surrounding environment increases in response to UV radiation or interspecific interaction with *M. aeruginosa.* The potential role of these OMVs to interact with neighboring cells or to promote cyanobacterial adaptation awaits further investigations.

## Author Contributions

RM provided study guidance, mentorship and critical editing of the manuscript. VZ, TS, NN, JG, and MMe performed the experiments, acquired and analyzed the data. VZ, TS, and NN performed the TEM analyses. MMa contributed with cyanobacteria strains and cultures. All authors contributed in part to writing and editing the manuscript and approved the final version.

## Conflict of Interest Statement

The authors declare that the research was conducted in the absence of any commercial or financial relationships that could be construed as a potential conflict of interest.
